# Rapidity of Genomic Adaptations to Prasinovirus Infection in a Marine Microalga

**DOI:** 10.3390/v10080441

**Published:** 2018-08-19

**Authors:** Sheree Yau, Gaëtan Caravello, Nadège Fonvieille, Élodie Desgranges, Hervé Moreau, Nigel Grimsley

**Affiliations:** 1 Integrative Biology of Marine Organisms Laboratory (BIOM), CNRS UMR7232, 66650 Banuyls-sur-Mer, France; sheeyau@gmail.com (S.Y.); caravello.gaetan@hotmail.com (G.C.); nadegefonvieille@yahoo.fr (N.F.); elodie.desgranges@microbiaenvironnement.com (É.D.); herve.moreau@obs-banyuls.fr (H.M.); 2 Sorbonne University, OOB, Avenue de Pierre Fabre, 66650 Banyuls-sur-Mer, France

**Keywords:** *Ostreococcus tauri*, Mamiellophyceae, Phycodnaviridae, resistance, karyotype, rearrangement, chromosome, specificity, host range, adsorption

## Abstract

Prasinoviruses are large dsDNA viruses commonly found in aquatic systems worldwide, where they can infect and lyse unicellular prasinophyte algae such as *Ostreococcus*. Host susceptibility is virus strain-specific, but resistance of susceptible *Ostreococcus tauri* strains to a virulent virus arises frequently. In clonal resistant lines that re-grow, viruses are usually present for many generations, and genes clustered on chromosome 19 show physical rearrangements and differential expression. Here, we investigated changes occurring during the first two weeks after inoculation of the prasinovirus OtV5. By serial dilutions of cultures at the time of inoculation, we estimated the frequency of resistant cells arising in virus-challenged *O. tauri* cultures to be 10^−3^–10^−4^ of the inoculated population. Re-growing resistant cells were detectable by flow cytometry 3 days post-inoculation (dpi), visible re-greening of cultures occurred by 6 dpi, and karyotypic changes were visually detectable at 8 dpi. Resistant cell lines showed a modified spectrum of host-virus specificities and much lower levels of OtV5 adsorption.

## 1. Introduction

Although the first prasinovirus was discovered in over 40 years ago [1], complete genomes of some of these abundant [2,3] large dsDNA viruses have become available only over the last 10 years [4,5,6,7,8,9]. Complete genomes of several of their tiny eukaryotic unicellular host algae have also been sequenced [10,11,12,13,14] and these species can be grown in culture, permitting genome-based physiological analyses of these ecologically important host-virus interactions to progress [15,16,17]. Using *Ostreococcus tauri virus 5* (OtV5), and its host microalga *Ostreococcus tauri,* we observed recently by transcriptomic analyses that in diurnal 12 h day/12 h dark laboratory-grown cultures, prasinovirus gene expression was low in the day but much higher in the night, the majority of cells lysing the following morning [15], giving a more complete overview of this host–virus life cycle. Viral gene transcripts were much more abundant in the night, when host cells remained intact and 4% of host genes were differentially expressed. Previous work showed that host resistance to viral lysis arises frequently in culture in different species of the Mamiellophyceae [18], and that this resistance was stable in culture over a period of 3 years, although a slight reduction in the fitness of these strains was detectable. Some of the clonally resistant lines produced were free of virus particles, while others produced viruses. Yau et al. [19] further investigated this phenomenon on a molecular level using a set of 38 independently produced virus-resistant clonal lines alongside eight control susceptible lines, maintaining all lines in parallel. Transcriptomic RNA-Seq analyses of the cell lines revealed differential expression (DE) of genes encoding glycosyltransferases, protein modifications, transporters, and DNA-modifying enzymes. Surprisingly, a disproportionate number of DE genes were localised on chromosome 19 and karyotypic analyses revealed that large-scale rearrangements of chromosome had occurred in OtV5-resistant lines. Many overexpressed genes were clustered in a 150 kb long region of chromosome 19 [19]. This work required the maintenance of clonal lines over a long period in order to obtain sufficient material for individual karyotype analyses and replicates for RNA-Seq comparisons, but in each clonal line resistance was acquired within a week of OtV5 inoculation. Here, we investigate the rapidity of and frequency of genetic changes occurring in the host alga more closely in the two weeks following OtV5-inoculation and test the effects resistance on viral specificity and binding on the set of previously produced resistant lines.

## 2. Materials and Methods

### 2.1. Culture Conditions

Host and viral cultures were grown in L1 medium (Bigelow Lab., NCMLA, Boothbay, ME, USA) under a 12 h light/12 h dark photoperiod at 80–100 μmol photon m^−2^ s^−1^ white light at 20 °C. The algal strain RCC4221 (Roscoff Culture Collection, Roscoff, France), a recently resequenced [11] clonal isolate of the wild-type sequenced *O. tauri* strain [10] that was previously known as RCC745, or at the time of isolation as OTH0595 [20,21], was used as the OtV5-susceptible control throughout. *Bathycoccus prasinos* RCC1105 and *Picochlorum* sp. RCC4223 were grown under identical conditions to those used for *O. tauri*. The virus strains used have been described previously [22] and were routinely used at MOI 5 on exponentially growing *O. tauri* cells to produce lytic infections. Host cell densities were measured using a BD FACSCanto II flow cytometer (BD Biosciences, Franklin Lakes, NJ, USA). Cellular autofluorescence of chlorophyll [23] was used to assess the number of viable algal cells. Specificity of viral strains to host strains was assayed by depositing 2 µL of viral lysate onto the host or test strain grown in a petri dish with L1 solidified with 0.15% agarose as previously described [22].

### 2.2. Karyotype Analysis

Algal cells were grown to a density of about 2 × 10^7^ cells∙mL^−1^, 8.7 × 10^7^ cells were pelleted by centrifugation (8000× *g* for 20 min), resuspended 100 µL TE buffer (10 mM Tris-HCl, 125 mM EDTA, pH 8), and embedded in plugs by mixing with an equal volume of molten low melting point agarose (1% in TE buffer precooled to 45 °C). Karyotype analysis was then done using pulsed field electrophoresis as previously described [19,24].

### 2.3. Virus Adsorption Assay

The protocol developed by Meints et al. [25] was adapted to suit our system. One-hundred mL of cultures of the different algae (*O. tauri* susceptible or resistant strains, *Bathycoccus* sp., or *Picochlorum* sp.) were grown up to around 2 × 10^7^ cells∙mL^−1^. Cultures were then centrifuged and pellets resuspended to obtain a final concentration of 10^9^ cells∙mL^−1^. Adsorptions of OtV5 to the different strains/species were assayed in 1 mL volumes of culture medium containing 10^9^ host cells and 10^7^ Plaque forming units (PFU) of virus (MOI 0.01). Samples were incubated for 30 min at 20 °C and the reactions were stopped by a centrifugation 10,000× *g* for 10 min. Supernatants were quickly transferred to fresh tubes and viral PFU measured by plating [4]. The data are expressed as the percentage of unadsorbed virus.

## 3. Results

### 3.1. Resistant Cells Grew 3 Days after Virus Inoculation

**Figure 1:**
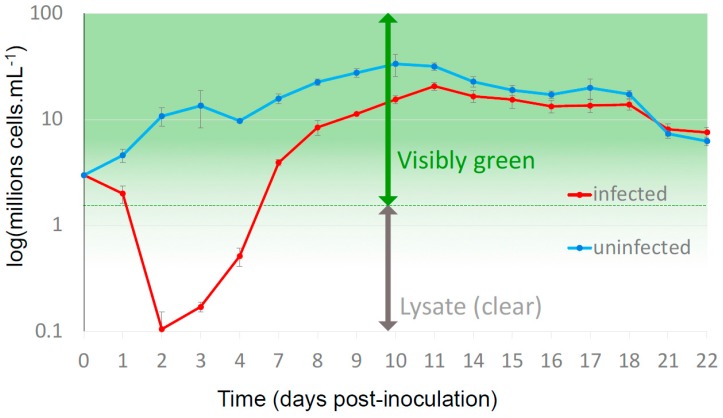
Host cell dynamics during a lytic infection. Each point represents an average from five independent cultures of *O. tauri*. Lysis occurs over two days following OtV5 inoculation, but at 3 days post-inoculation (dpi) OtV5-resistant cells observed by flow cytometry were growing, visible re-greening of the cultures occurring by 5 to 6 dpi.

After inoculation with OtV5, the number of viable cells of *O. tauri* decreased from 2.98 × 10^7^ cells∙mL^−1^ at the time of inoculation to 2.00 × 10^7^ ± 3.76 × 10^6^ cells∙mL^−1^ (*n* = 5) at 24 hpi (hours post-inoculation) and 1.05 × 10^6^ ± 4.76 × 10^5^ cells∙mL^−1^ at 48 hpi. At the same time, cell densities increased in control cultures from 2.98 × 10^7^ cells∙mL^−1^ to 4.59 × 10^7^ ± 6.34 × 10^6^ cells∙mL^−1^ (*n* = 3) at 24 hpi and 1.07 × 10^8^ ± 2.11 × 10^7^ cells∙mL^−1^ at 48 hpi, consistent with about one cell division per day. However, on the 3rd day (72 hpi), the average number of viable cells in infected cultures increased (Figure 1), with an overall (*n* = 5) mean of 1.70 × 10^6^ ± 1.78 × 10^5^ cells∙mL^−1^ and by the 4th day all of the 5 cultures were re-growing despite the presence of an excess of infectious virus particles (as measured by PFU plated on the susceptible host strain). Moreover, duplicate aliquots of these regrown virus-inoculated cultures did not lyse compared to noninfected controls when re-infected with OtV5. These cells were thus virus-resistant. Furthermore, the virus-resistant cells grew at more than 1 division per day over the following 3 days (Figure 1), confirming that their fitness must be close to that of the wild-type strain. In this experiment, the inoculated cultures appeared as cleared lysates for a period of about 4 days before they started to re-green, in agreement with previous observations [18].

### 3.2. Evidence for Chromosomal Rearrangements within 8 Days

**Figure 2:**
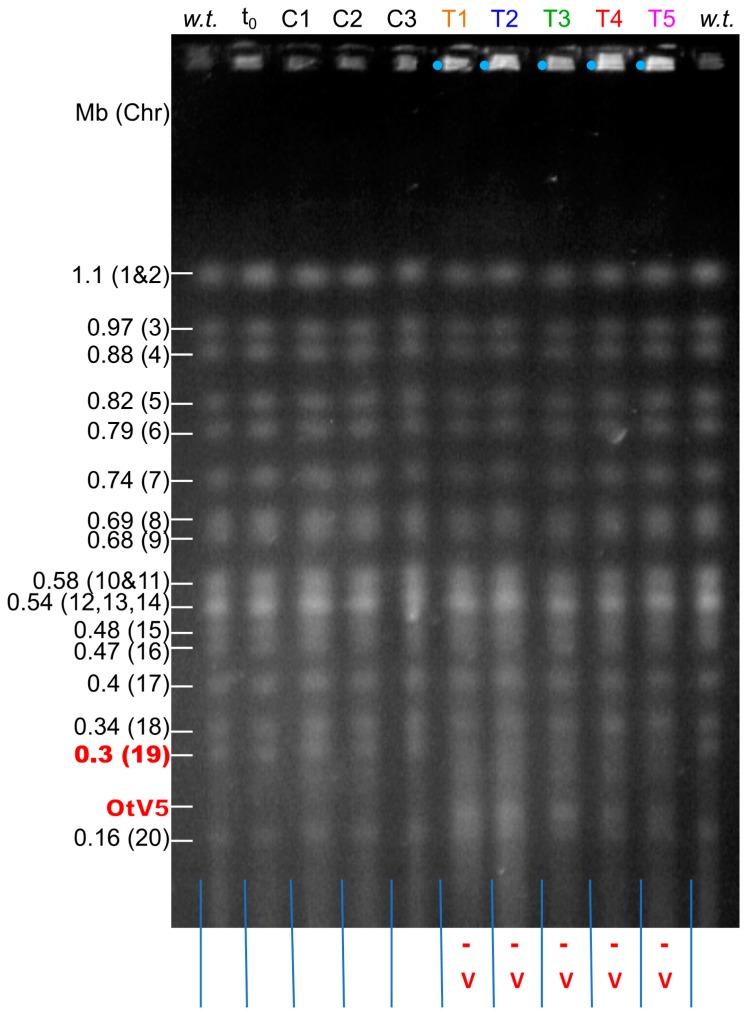
Karyotypes of *O. tauri* cultures visualised by pulsed field gel electrophoresis (PFGE) separation of the chromosomes. The sizes and positions of *O. tauri* chromosomes are indicated on the left. Chromosome 19 (red numbers) was not visualised in freshly OtV5-resistant test cultures (gel tracks indicated with red annotations at the bottom). ***w.t*.**: wild-type *O. tauri* RCC4221 from the original culture used before inoculation. **t_0_**: wild-type *O. tauri* RCC4221 from the RCC4221 culture at the start of the experiment. **C1**, **C2** and **C3**: mock-inoculated Control cultures at 8 days post-inoculation (dpi). **T1–T5**: Test cultures inoculated with OtV5 at MOI 5 (colours correspond with those in Figure 3 below), 8 dpi. **-**: absence of chromosome 19 at its expected mobility (312 kb) in test cultures. **v**: presence of the OtV5 viral genome (184 kb long) in inoculated cultures. **Blue dots**: wells with higher fluorescence.

Since Yau et al. [19] observed that 34 of 36 independent OtV5-resistant clonal lines showed chromosomal rearrangements during a period of co-evolution of host and virus over several months, here we aimed to determine if chromosomal changes occurred as soon as possible following inoculation with OtV5. Pulsed field gel electrophoresis (PFGE) was used to examine the karyotypes of individual cultures as soon as sufficient cells were present to perform such an analysis from the cultures mentioned above (Section 3.1). Eight days after inoculation with OtV5, five infected (Test) cultures and three noninoculated (Control cultures) were used to prepare intact chromosomal DNA. Their karyotypes were then compared by PFGE. In the five test lines, as expected, the linear OtV5 genome could be observed at its expected size of 184 kb with a band intensity similar to the *O. tauri* chromosomes indicating the virus is still present in the cultures at a high virus to cell ratio. However, in these cultures, chromosome 19, expected at about 300 kb [19], was no longer visible (Figure 2), but there was increased fluorescence in the gel wells, again suggesting the presence of circular or very large DNA intermediates [19]. No visible changes in the sizes of other chromosomes were evident.

**Figure 3:**
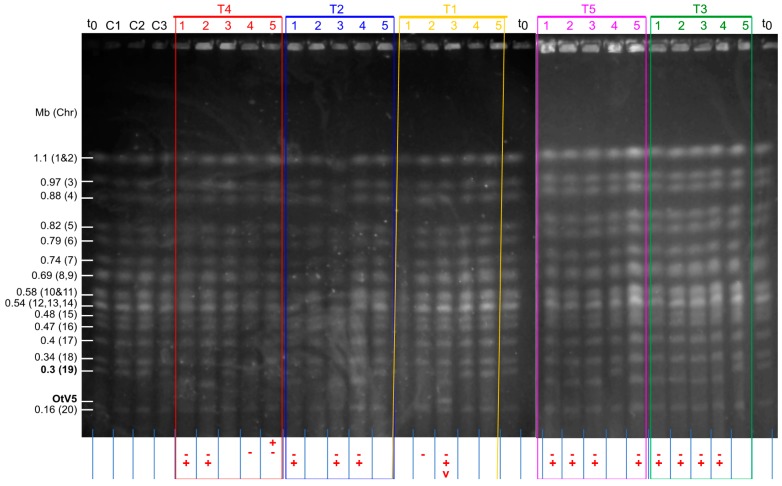
Karyotypes of clonal lines *O. tauri* visualised by PFGE separation of the chromosomes. The figure is a composite of two separate gels. **t_0_**: wild-type *O. tauri* RCC4221 the start of the experiment. **C1**, **C2**, and **C3**: clonal lines established from mock-inoculated control cultures. **T1–T5**: Five independent clonal lines were established from each of the Test (T1–T5) OtV5-resistant cultures shown in Figure 2, i.e., five lines from each of the five cultures (labelled and boxed with similar colours in both figures). Annotations underneath the gel tracks show which tracks have bands with altered mobilities. **-**: absence of chromosome 19 at its expected mobility (0.3 Mb, indicated position in bold on the left) in test cultures. **+**: presence of a chromosomal band not detected in Control cultures. **v**: presence of the OtV5 viral genome (184 kb long) in inoculated cultures.

Clonal lines were then established from all cultures by spread plating and randomly picking individual colonies into fresh liquid medium. Five clonal lines were picked from each of the test OtV5-resistant cultures and 1 clonal line from the control susceptible cultures. Their karyotypes were then compared by PFGE (Figure 3).

None of the clonal lines from the control cultures showed a different karyotype to the wild-type *O. tauri*. In the clonal OtV5-resistant lines, chromosome 19 was now resolved as a band detectable by PFGE in 22 of 24 resistant clones (one of the karyotype profiles was not resolved). Fourteen resistant lines showed a decrease in size of the SOC, one line showed an increase in size and the remaining seven showed no visible change compared to the wild-type. Importantly, the sizes of the chromosome 19 in clones coming from the same parent resistant culture always varied between clones, indicating that the parent resistant cultures consisted of cells with a mixture of SOC sizes.

### 3.3. Acquisition of Resistance Is Frequent

**Figure 4:**
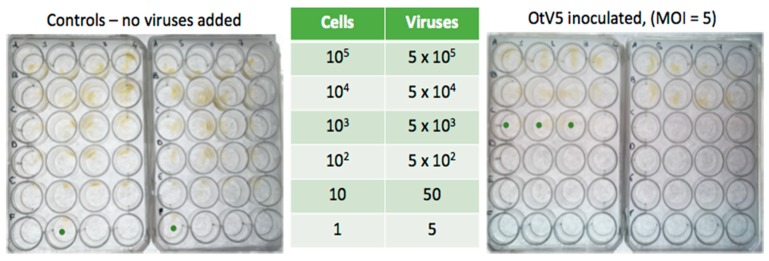
(**Left**): uninfected cultures. (**Right**): cells inoculated with OtV5 at MOI 5, as shown in the table. Each of the 16 columns of wells is a separate culture, diluted as shown in the table in rows top (least dilute) to bottom (most dilute). Green dots indicate the most dilute step of the series in which host cell growth was visible in a proportion of the cultures.

In order to make an approximate estimation of the minimal number of cells required for resistance, 16 independent cultures were used to make a set of dilution series’ for inoculations, eight controls with L1 medium added and eight with a suspension of OtV5 in L1 medium. The plates were then incubated for 20 days to permit growth of host cells and viruses (Figure 4).

In controls 2/8 cultures diluted to an average of 1 cell per culture well grew, and 8/8 cultures started with 10 cells grew, whereas in OtV5-inoculated cultures about 37% of the cultures started with 1000 cells in *O. tauri* became resistant, and 10^4^ cells gave rise to resistant lines in 8/8 independent cultures. Thus, 1 in 10^3^–10^4^ cells gave rise to a resistant line in these cultures.

### 3.4. OtV5-Resistant Strains Showed a Wide Spectrum of Viral Resistance

**Figure 5:**
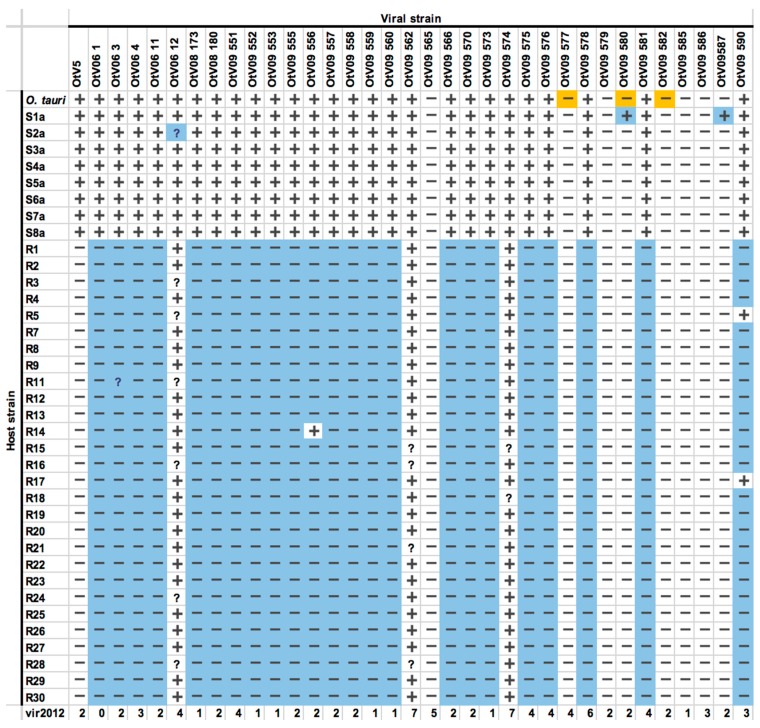
Altered spectrum of virus resistance in OtV5-resistant *O. tauri* lines. **+**: complete or almost complete host cell lysis **-**: no or little host cell lysis. **?**: not possible to determine. Orange background cell: result on susceptible *O. tauri* control differed from previous observation [22]. Blue background cell: result that differed to the null hypothesis that the susceptibility of this strain would be the same as that previously observed (S1a to S8a controls and those of a past paper [22]). Note that OtV5 is not highlighted since these lines were selected for their resistance to this virus. **S**: susceptible clonal line [19]. **R**: resistant clonal line [19]. **vir2012**: The number of independently isolated wild-type *O. tauri* strains on which the viral isolate shown was noted as virulent out of 12 tested previously [22] (excluding the strain on which the virus was isolated).

In order to test whether the acquisition of resistance led to an altered specificity range we used host and viral strains produced in previous studies. Many viruses of *O. tauri* have been isolated from samples collected from different locations or on different sampling dates, and their host-virus specificities have been tested [22,26]. We tested the virulence of these independently isolated viruses on the set of 8 OtV5-susceptible control lines and 28 of the OtV5-resistant clonal lines previously produced by experimental co-evolution of host and virus [19]. Overall, OtV5-resistant lines showed an increased spectrum of resistance to viruses that were infectious in the original *O. tauri* strain (Figure 5).

Resistance to OtV5 in the evolved clonal lines also conferred resistance to 89% (24/27) of other independently isolated viruses [22] of the original *O. tauri* virus-susceptible strain.

### 3.5. OtV5-Resistant Strains Do Not Adsorb OtV5

**Figure 6:**
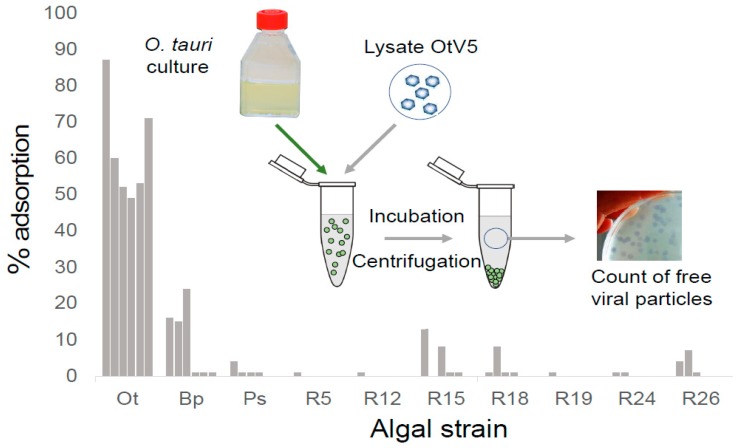
Acquired resistance reduces adsorption of OtV5. Ten algal strains (abscissa) were each assayed six times (grey bars) for their ability to bind to OtV5. **Ot**: control susceptible wild-type *O. tauri* RCC4221, **Ps**: *Picochlorum* sp. RCC4223, **Bp**: *Bathycoccus* sp. RCC4222, **R**: OtV5-resistant line [19].

Naïvely, since the vast majority of cells were asymptomatic in OtV5-resistant lines exposed to OtV5, we might expect that OtV5 particles no longer bind to resistant cells. We tested this hypothesis using adsorption assays to determine the number of PFU remaining in an OtV5 lysate after incubation together with different resistant or susceptible lines of *O. tauri* and related species of microalgae to measure the number of infectious particles (number of PFUs) which do not adsorb on *O. tauri* resistant cells (Figure 6). Control susceptible *O. tauri* cells adsorbed 49–87% of the viable particles present in the suspension, whereas the nonhost species *Bathycoccus* sp. [14] and *Picochlorum costavermella* [27] adsorbed 1–24% and 0–4%, respectively (variations in 6 replicates). There was thus some adsorption to the phylogenetically more closely related *Bathycoccus* sp. (Mamiellophyceae) ancestral species but little or none to the phylogenetically more distant green alga *P. costavermella* (Trebouxiophyceae). Seven independently isolated OtV5-resistant clones [19] showed much reduced adsorption to *O. tauri* cells, in support of the hypothesis that at least some of the resistance observed is due to lack of adsorption to these cells.

## 4. Discussion

### 4.1. Is Resistance Induced?

We observed that about 5 × 10^6^ cells∙mL^−1^ were growing at 4 dpi (Figure 1). A freshly cloned culture of *O. tauri* was used, an MOI of five viral particles was present at the start of the experiment, and might hypothesize that resistance may have been induced in the night the first infection cycle, since most of the host and viral changes in gene expression occurred at that stage [15]. By “induced” we mean that some process that leads to cellular viral resistance has been initiated by the infecting virus. Assuming one division per day, resistance may thus have been induced in about 3.0 × 10^5^ cells∙mL^–1^ in the 3.0 × 10^7^ cells∙mL^−1^ used for inoculation (i.e., about 3 in 10^4^ cells). This figure is much higher than the spontaneous mutation frequency of 4 × 10^−10^ mutations per nucleotide per generation in *O. tauri* [28]. Independently, we showed clearly in a subsequent experiment (above 3.3) that about 10^3^–10^4^ cells were necessary for resistance, confirming this rough calculation.

While our results strongly favour the notion that resistance is induced, we have also observed in this study and in previous work [19] that OtV5-resistance is strongly correlated with an increased rate of spontaneous large rearrangements concentrated on the SOC. These changes may correspond mainly to deletions and duplications occurring in the genetic material on this chromosome, perhaps arising because of the selective pressure to overexpress some genes affecting resistance to OtV5, but this interpretation remains to be investigated experimentally. Since the SOC represents only 2% of the complete genome, a higher mutation rate was likely not detectable in this chromosome using short read technology employed for measuring the spontaneous mutation rate by mutation accumulation [28] because of repeated DNA in the SOC. Moreover, the spontaneous mutation rate measured was of single nucleotide and short insertion-deletions (indels) and thus could not take into account large indels or rearrangements. In wild-type strains, the SOC is variable in size and composition [29,30], suggesting that the strong selective pressure arising from dense prasinovirus populations in coastal environments [31] also favours genetic hypervariability in the SOC. Given that we were unable to measure the spontaneous rate in the SOC, we cannot rule out an alternative hypothesis, that a small proportion of cells in the population always contain some level of ongoing SOC rearrangements that are usually lost because of their reduced fitness. Intuitively this hypothesis seems less likely, because the control *O. tauri* RCC4221 karyotype has been stable through subculturing over many years [28] and there is little difference in the fitness between susceptible and resistant strains [18,32].

### 4.2. Genomic Rearrangements Occur Rapidly

In OtV5-resistant cultures at 8 dpi, chromosome 19 could not be seen at the expected position on PFGE gels (Figure 2) consistent with these cultures containing a mixture of lines with different rearrangements of chromosome 19 (Figure 3).

Thus, this study showed firstly, that SOC rearrangements were closely linked in time to OtV5 infection and acquisition of resistance, and secondly, that a variety of rearrangements reliably occurred in the culture population. By contrast to Yau et al. [19] where resistant lines had been propagated over months, variability in SOC size was lower, with a larger proportion of resistant clones showing no visible change in SOC size, and no changes were evident in other chromosomes. This suggests continued host-virus interactions produces larger variability as a result of an accumulation of mutations over time. As PFGE is not able to reveal small indels or rearrangements, we cannot rule out that smaller changes may have occurred in lines with no apparent change in SOC size, so it remains uncertain whether these rapid rearrangements in the SOC are necessary for resistance or whether they are a by-product of contact with OtV5. If resistance is always linked to rearrangement of the chromosome 19, the appearance of a variety of SOC types was as expected given the frequency of appearance of resistant cell lines measured in Section 3.3. Given that on average 3 cells in 10^4^ would be expected to become resistant, and the inoculated culture was at about 3 × 10^7^ cells∙mL^−1^, then we might expect there could in theory be up to 10^4^ different resistant clones present. However this will certainly be an overestimate, as only the fastest-growing cell lines would reach the cell density required to produce enough cells for PFGE analysis, and eventually the fastest growing variants would dominate the culture population.

### 4.3. Acquired Resistance Shows a Broad Specificity Spectrum

The much increased spectrum of resistance observed (Figure 5) to other viral strains suggests that a global adaptive response has been induced, and concurs with differential expression of many genes [19], those on chromosome 19 being particularly important. The three viral strains (OtV06_12, OtV09_562, and OtV09_574) still able to lyse the majority of the OtV5-resistant lines (Figure 5) were shown by Clerissi et al. [22] to be among the most virulent in terms of the large proportion of independently isolated host strains (up to 85% of *O. tauri* strains) they lyse. Furthermore, these three viral strains were of different DNA polymerase haplotypes when compared to the other viral strains which can no longer lyse the OtV5-resistant lines [22]. This is consistent with observation that more closely related viruses, based on similarity in taxonomic marker genes, are able to lyse a similar range of host strains [22,26]. Accordingly, resistance to one virus strain would be expected to confer resistance to genetically related strains. The effectiveness of the barrier to infection in resistant strains is witnessed by the very low level of OtV5 viruses adsorbing to them (Figure 6), since even the nonhost species *Bathycoccus* sp. RCC4222 bound a higher proportion of virions than OtV5-resistant host *O. tauri* cells (Figure 6). We were surprised to find that some of the viruses (on average 10%) adsorbed to *Bathycoccus* sp., which is not susceptible to any of them [22]. *Bathycoccus* spp. are relatively abundant in marine environments [33] and might thus compete for adsorption of *Ostreococcus* sp. viruses.

Resistance to such an extensive set of independently isolated viruses suggests that *O. tauri* has a fast adaptive mechanism for resistance to viruses that permits induction of a specialised set of genes, many of which were probably regrouped on chromosome 19, that may have been subject to the strong selective pressure of abundant prasinoviruses in eutrophic coastal waters. Bellec et al. [31] first observed that coastal lagoons waters are much more densely populated with prasinoviruses than offshore locations. Further work will aim to elucidate the evolution and molecular bases of resistance or susceptibility to viruses in this class of algae that have been so successful and pervasive in aquatic environments.
